# Molecular evolution and structural analyses of proteins involved in metabolic pathways of volatile organic compounds in **Petunia hybrida* (Solanaceae)*

**DOI:** 10.1590/1678-4685-GMB-2022-0114

**Published:** 2022-12-16

**Authors:** Lucas C. Beltrame, Claudia E. Thompson, Loreta B. Freitas

**Affiliations:** 1 Universidade Federal do Rio Grande do Sul, Departamento de Genética, Laboratório de Evolução Molecular, Porto Alegre, RS, Brazil.; 2 Universidade Federal de Ciências da Saúde de Porto Alegre, Departamento de Farmacociências, Porto Alegre, RS, Brazil.

**Keywords:** Volatile organic compounds, CFAT, BPBT, Solanaceae, molecular evolution, protein structure

## Abstract

The association between plants and their pollinators is essential for increasing the diversity in angiosperms. Morphological and physiological traits, mainly floral scent, can influence the pollination dynamics and select pollinators for each plant species. In this work, we studied two proteins involved in producing volatile organic compounds in plants, *conyferyl alcohol acyltransferase* (CFAT) and *benzoyl-CoA:benzyl alcohol/phenyl ethanol benzoyl transferase* (BPBT) genes. We aimed to understand these proteins with respect to evolutionary and structural aspects and functions in Solanaceae using phylogenetic methods and comparative molecular modeling. We used Bayesian inference to describe the proteins’ evolutionary history using *Petunia x hybrida* as a query to search for homologs in the Solanaceae family. Theoretical 3D models were obtained for both proteins using *Panicum virgatum* as a template. The phylogenetic tree included several different enzymes with diverse biological roles in Solanaceae, displaying the transferase domain. We identified only one sequence of CFAT in the databases, which belongs to *Petunia x hybrida,* and found several BPBT sequences from the genera *Nicotiana, Solanum,* and *Capsicum*. The 3D structures of CFAT and BPBT have two different domains, and we have identified the amino acid residues essential for the enzymatic activity and interaction with substrates.

## Introduction

The interaction between plants and their pollinators is responsible for a large portion of morphological and structural diversity in angiosperms (Lunau, 2004), which, in some cases, drove their speciation (Fregonezi et al. 2013). Formerly defined as a set of convergent floral traits (Faegri and van der Pijl, 1979), the floral syndromes are associated with specific pollinator groups (Fenster et al., 2004; Etcheverry and Alemán, 2005) and allow to predict which group will be attracted to a given plant species (Schiestl and Johnson, 2013). Many floral traits, such as shape, size, petal color, nectar production, and fragrance, can play a critical role in the attraction and frequency of pollinators and influence the quality of pollinator’s visits. In addition, several volatile compounds produced mainly in petals are involved in the attraction of pollinators (Raguso, 2001; Knudsen and Gershenzon, 2006; Knudsen *et al.,* 2006). These aromatic stimuli are learned more quickly than visual clues (Arenas and Farina, 2014) and may differentially attract certain pollinator species (Huber et al., 2005; Klahre et al., 2011), especially insects.

The floral scent is essential compared to visual cues in pollination systems involving plants and nocturnal insects (Rachersberger et al., 2019). The floral scent profile is also associated, in general, with pollinator species in particular. For example, plants that emit high levels of benzenoid compounds are related to moths (e.g., Dobson, 2006). Moreover, some species are known for producing deterrent compounds such as isoeugenol and benzyl benzoate to avoid florivory by insects and other animals (e.g., Dudareva et al., 2013). Even some bees are attracted by pollen scent that contains certain specific compounds (e.g., Rodrigues et al., 2018).

The Solanaceae family encompasses species with tremendous economic and ecological significance, including several cultivated species used in food and ornamentation (Olmstead et al., 2008). Different pollination syndromes are observed among Solanaceae species, and morphological traits are relevant to understanding the evolutionary history of these species and predicting pollinator-plant relationships (Knapp, 2010). The floral volatile organic compounds (VOCs) are strong cues for pollinators and herbivores in Solanaceae (Kessler, 2012).

The *Petunia* genus is a charismatic group of wild species distributed in southern South America and the ornamental and widely known *Petunia hybrida* (Stehmann et al., 2009). The *P. hybrida* is one of the most profitable cultivated species globally and a model for plant genetics and physiological studies (Vandenbussche et al., 2016). This commercial plant is an artificial hybrid developed in the 19^th^ century from crossings between *P. axillaris,* a white-flowered and moth-pollinated plant, and the pink and bee-pollinated *P. interior* species (Segatto et al., 2014).

This study focused on two proteins involved in metabolic pathways of production and emission of VOCs, the benzoyl-CoA:benzyl alcohol/phenyl ethanol benzoyl transferase (BPBT; EC 2.3.2.196) and the coniferyl alcohol acyltransferase (CFAT; EC 2.3.1.84). BPBT is involved in VOCs production, such as phenyl-ethyl benzoate and benzyl benzoate; meanwhile, CFAT converts coniferyl alcohol into coniferyl acetate, a precursor of eugenol and isoeugenol VOCs (Dudareva et al., 2013; Amrad et al., 2016). BPBT and CFAT proteins are members of the large enzymatic family named BAHD (an acronymous of the first four characterized proteins in this group). BAHD are acyltransferases and utilize CoA thioesters to catalyze the formation of several plant secondary metabolites (D’Auria, 2006), including VOCs. BPBT and CFAT are represented by one copy in *P. hybrida* (Dexter et al., 2007).

Previous studies showed that the proteins from the BAHD family fall broadly into five main clades (Tuominen et al., 2011) and contain two conserved protein motifs, HxxxD and DFGWG, which have facilitated *in silico* identification of BAHD acyltransferases from available genome sequences (Luo et al., 2007; Yu et al., 2009; Tuominen *et al.,* 2011). The HxxxD motif is involved in the catalytic activity, whereas DFGWG has a structural role that promotes the stable folding of the protein (Ma et al., 2005). However, even sharing the active motifs and included in the same enzymatic family, BPBT and CFAT proteins can perform many different reactions, acting with a broad spectrum of substrates with low specificity (Wang et al., 2021).

Our main aim was to better understand the evolutionary history of BPBT and CFAT proteins in the Solanaceae family using phylogenetic methods and comparative molecular 3-D modeling with an emphasis on *P. hybrida* sequences. In addition, we based our study on the hypothesis that these proteins have evolved by convergence regarding the VOC type that each one produces and the preferential group of pollinators attracted by the plant species.

## Material and Methods

### Sequences retrieval and alignment

We used BLAST searches with default parameters to obtain homologous protein sequences (PhBPBT and PhCFAT) from the public sequence database National Center for Biotechnology Information (NCBI Resource Coordinators, 2016; NCBI, 2022) using *Petunia hybrida* BPBT and CFAT protein sequences as queries (GenBank ID: AAU06226.1 and ABG75942.1, respectively). We analyzed the protein domains using the *hmmscan* tool in HMMER (Finn et al., 2011; HMMER, 2020) software against Pfam (Finn *et al.*, 2016; Pfam, 2022) database with default parameters. The next step was to identify the HMM (Hidden Markov model) profile and generate a local database including all sequences with a similar profile using the *hmmsearch* tool from HMMER against the UniProtKB database (The UniProt Consortium, 2017) with default parameters. We restricted the results to include only sequences from Solanaceae species; as a result, we retrieved all protein sequences with the same HMM profile.

We used BLAST+ (Camacho et al., 2009) software to format a local database using the sequences previously identified and searched for homologs using a specific cutoff (e-value ≤ 10^-6^). We downloaded the sequences in FASTA format from the list of sequences that matched these criteria and excluded all partial sequences. Subsequently, we performed the alignment using the PRANK (Löytynoja and Goldman, 2005; PRANK, 2022) software with default parameters. The alignment was manually edited using AliView software (Larsson, 2014; AliView, 2021), excluding ambiguous positions. 

### Molecular genealogy

We used ProtTest (Abascal et al. 2005) with default settings to choose the best-fit models of amino-acid replacement based on AIC (Akaike’s information criterion), BIC (Bayesian information criterion), AICc (AIC with a correction for finite sample sizes), and DTC (Decision Theory criterion) criteria. We obtained a phylogenetic tree for acyltransferases (including the BPBT and CFAT proteins) using the probabilistic Bayesian inference method in MrBayes software (Ronquist et al., 2011; MrBayes, 2019) with default parameters and the best evolutionary model. We ran four chains for 2,000,000 generations and discarded 25% of the genealogies as burn-in. 

### Structural analyses

To theoretically model the previously published BPBT and CFAT proteins from *P. hybrida* (PhCFAT GenBank ID: ABG75942.1; PhBPBT, GenBank ID: AAU06226.1), we downloaded the FASTA sequences and used them as queries for searching homologs with identified three-dimensional structures using the BLAST tool (Altschul et al., 1990) against the Protein Data Bank (Berman *et al.,* 2000; PDB, 2022).

We used the resolution equal to or higher than 2.0 Å, identity higher than 25 %, and high coverage as the criteria to choose the best template for the comparative molecular modeling (Sánchez and Sali, 1997; Wiltgen, 2018). In addition, we ran the MAFFT software (Katoh and Standley, 2013; MAFFT, 2021) with default parameters to align queries and templates and inspect the coverage among them. After identifying the best templates, we used MODELLER software (Sali and Blundell, 1993; Webb and Sali, 2014; MODELLER, 2022) to model the three-dimensional structures of PhBPBT and PhCFAT proteins.

We used the PROCHECK (Laskowski et al., 1993; PROCHECK, 2010) and Verified 3D (Lüthy et al., 1992) software to verify each generated model’s quality and stereochemical parameters. An essential criterion for choosing the best model is the number of residues within the most favored and additional allowed regions identified on the Ramachandran plots (Ramakrishnan and Ramachandran, 1965; Morris et al., 1992) generated by PROCHECK. These regions are based on plotting the dihedral angles ψ (psi) and Φ (phi) of each residue of the protein main chain evaluating which bonds between residues are spatially and structurally favored. The lowest number of residues with geometric distortions is also helpful in choosing the best model. We obtained the theoretical isoelectric point using the ExPASy Tools software (Gasteiger et al., 2005; ExPASy, 2020). Finally, we generated figure models using the PyMol software (2022), including each protein’s electrostatic potential. 

## Results

### Sequence retrieval and alignment

The search for BPBT and CFAT proteins recovered enzymes with a transferase domain starting at the 11^th^ site and ending at the 441^st^ site for BPBT and from the 6^th^ to 438^th^ sites for CFAT. We found 807 sequences per query from Solanaceae taxa, resulting in 502 and 501 homologous sequences for PhBPBT and PhCFAT proteins, respectively, after filtering by HSP (high scoring pairs) with a permissive e-value = 10 (Table S1). The same proteins were simultaneously recovered with both queries when all sequences were compared. All repeated IDs were then discarded. We applied a threshold of at least 25% identity to select the final data set and excluded partial sequences, resulting in 335 sequences identified in the UniProtKB AC/ID database. These sequences were aligned, and all ambiguous positions were manually excluded. The final alignment included 1,578 amino acid residues. 

### Phylogenetic tree construction

**Figure 1 -:**
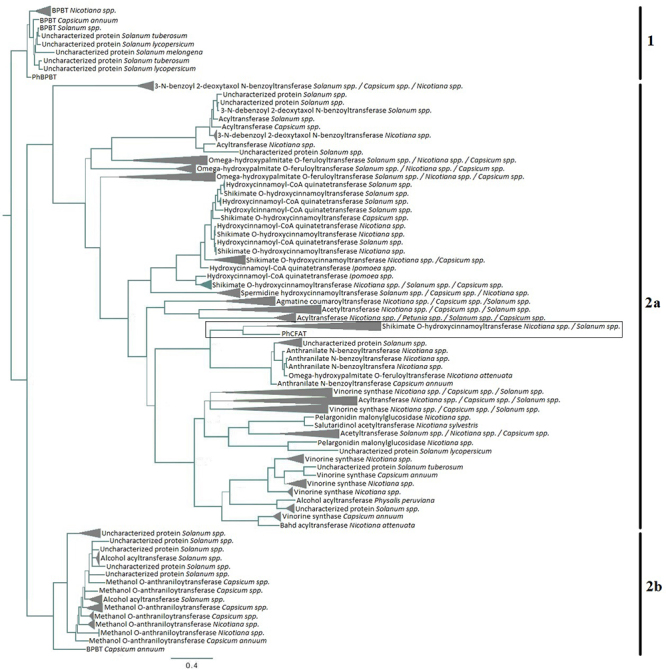
Solanaceae protein with transferase domain simplified phylogenetic tree generated using Bayesian Inference and JTT + I +G as evolutionary method and model, respectively. The analysis included BPBT and CFAT sequences. Branches with more than one sequence from the same genus were collapsed and indicated with *spp*. Wider branches indicate PP > 0.80 while narrowed branches had PP < 0.80. The rectangle highlights PhCFAT relationships.

The best-fit evolutionary model for this data set was J.T.T. + I + G [Jones-Taylor-Thornton (Jones et al., 1992) + Invariant sites + Gamma distribution]. A phylogenetic tree was obtained, representing all Solanaceae acyltransferase proteins with high posterior probability values for the branches (mainly PP> 0.80), especially ancient events. The tree had two main clades grouping different proteins with the transferase domain (Figure 1). 

Clade 1 included BPBT sequences from *Nicotiana*, *Capsicum*, *Solanum*, the *Petunia hybrida* BPBT (PhBPBT), and other proteins with no characterized function from *Solanum* species. PhBPBT was the sister group of the remaining sequences, and all BPBT sequences from *Nicotiana* species were grouped (PP = 1.0). Only one BPBT sequences was not included in this clade (an isoform observed in *Capsicum annum,* see below). The uncharacterized sequences of *Solanum* species grouped with *Solanum* BPBT (PP = 0.93) suggesting that even displaying some differences among species, these proteins are at least BPBT-like. The *C. annum* BPBT sequence was the sister group of *Solanum* sequences. Different insect groups pollinate these species, and the relationships among BPBT sequences were compatible with the genera phylogenetic position.

Clade 2 included PhCFAT, a *C. annuum* BPBT isoform, multiple transferase proteins involved in different metabolic pathways, such as phenylpropanoids’ biosynthesis (mainly hydroxycinnamic acid amides - HCAAs), and several uncharacterized proteins from wild and cultivated species in *Solanum, Capsicum, Nicotiana, Ipomoea, Lycianthes, Datura,* and *Physalis* genera. This clade was subdivided into two main subclades. The subclade 2a grouped proteins with varied functions disposed on minor clades mainly based on their specific activity. Some of these proteins indicated ancient duplications, mainly in *Nicotiana*, *Solanum*, and *Capsicum*, the most studied genera. Subclade 2b encompassed BPBT isoform of *C. annuum* as the sister group of *Solanum* uncharacterized proteins and *Solanum*, *Capsicum,* and *Nicotiana* methanol and alcohol transferases. 

### Protein 3D structure

Using the previously published PhBPBT and PhCFAT protein sequences as queries to search for homologs with three-dimensional (3D) structures previously determined, we obtained nine hits for BPBT and eight for CFAT. Based on the criteria of highest coverage, identity, and resolution, we selected the crystal structure of *Panicum virgatum* shikimate hydroxycinnamoyl transferase (HCT) in complex with Coa and P-coumaroyl-shikimate (PvHCT2a; PDB ID: 5FAL, chain A; Eudes et al. 2016b) as a template for modeling BPBT and CFAT proteins in *P. hybrida*. The selected template had a resolution of 1.9 Å, with 30% of identity and 96% of coverage to BPBT, 29% of identity and 95% of coverage to CFAT. As we observed that PhCFAT is evolutionarily close to a monophyletic group of HCT from different species (Figure 1), it is justified to use a shikimate hydroxycinnamoyl transferase to model both CFAT and BPBT proteins.

**Figure 2 -:**
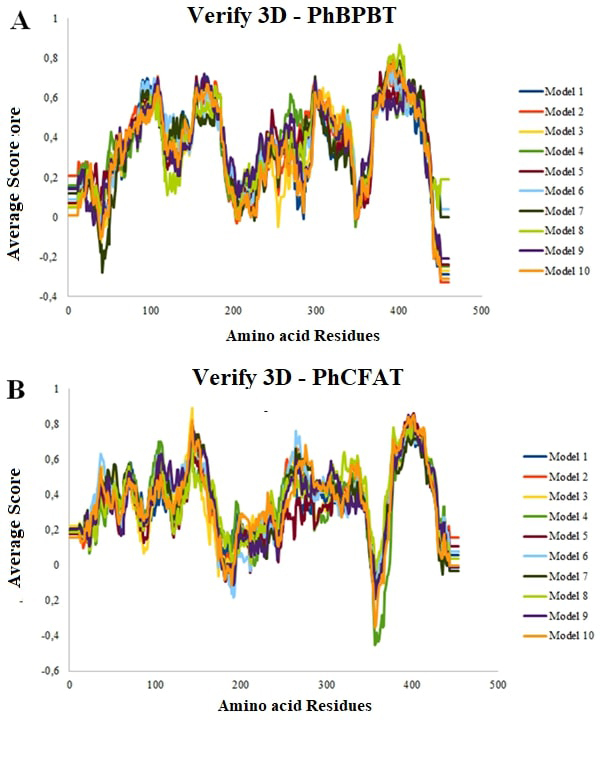
Evaluation of theoretical 3-D models using Verify 3D for *Petunia hybrida.* (A) PhBPBT protein sequence; (B) PhCFAT protein sequence.

The comparative molecular modeling generated 10 models for PhBPBT and PhCFAT proteins (see Table 1 for the main stereochemical data for all models). First, we evaluated the Ramachandran plot obtained in the PROCHECK to obtain information about the residues within the most favored, additional allowed, generously allowed, and disallowed regions (Table 1). All models showed more than 95% of the amino acid residues in the most favored and additional allowed regions. According to the stereochemical analyses, we chose model 8 (Figure 2A) and model 3 (Figure 2B) as the best models for PhBPBT and PhCFAT proteins, respectively, as both showed high and positive scores for the sum of the 3D profile based on the Verify 3D (2021) analysis. Additionally, the G-factor values were -0.20 for PhBPBT and -0.29 for PhCFAT, indicating minor and insignificant geometric troubles in the model configuration.

**Table 1:** Stereochemical data for all 3-D generated models for PhBPBT and PhCFAT proteins.

BPBT										CFAT									
Model	Ramachandran				Distortions				G-factors	Model	Ramachandran				Distortions				G-factors
	F+AF	F	AF	GA	D	TL	Plan	DA	Overall		F+AF	F	AF.	GA	D	TL	Plan	DA	Overall
1	97.9	89.4	8.5	1.5	0.5	21	1	17	-0.20	1	97.3	86.5	10.8	1.8	1.0	44	0	30	-0.33
2	98	87.9	10.1	0.8	1.3	16	0	12	-0.17	2	97.3	86.5	10.8	1.5	1.2	25	0	24	-0.29
3	96.9	86.6	10.3	2.6	0.5	18	0	11	-0.20	3	97.2	86.0	11.2	1.5	1.2	29	0	21	-0.29
4	98.4	88.9	9.5	1.0	0.5	18	0	12	-0.20	4	96.5	86.0	10.5	2.5	1.0	35	0	20	-0.30
5	97.9	87.6	10.3	1.0	1.0	24	0	22	-0.21	5	96.7	86.5	10.2	1.8	1.5	38	0	26	-0.28
6	97.7	88.9	8.8	1.0	1.3	22	1	14	-0.20	6	97.2	86.2	11.0	2.0	0.8	33	0	23	-0.31
7	98.4	89.9	8.5	0.8	0.8	20	2	14	-0.18	7	97.0	85.5	11.5	2.2	0.8	35	0	29	-0.32
8	98.2	89.4	8.8	1.3	0.5	25	1	16	-0.20	8	95.3	86.8	9.5	2.2	1.5	39	0	23	-0.31
9	98.2	87.4	10.8	0.5	1.3	20	1	20	-0.21	9	97.2	86.2	11.0	1.2	1.5	30	0	18	-0.27
10	97.9	89.4	8.5	1.5	0.5	21	1	17	-0.20	10	97.5	87.0	10.5	1.0	1.5	34	0	25	-0.32

F = % residues in most favored regions; AF = % residues in additional allowed regions; GA = % residues in generously allowed regions; D = % residues in disallowed regions; TL = number of bond lengths distortions within main-chain; Plan = number of distortions in planar groups; DA = number of bond angle distortions within the main-chain. Bold values indicate the best model.

**Figure 3 -:**
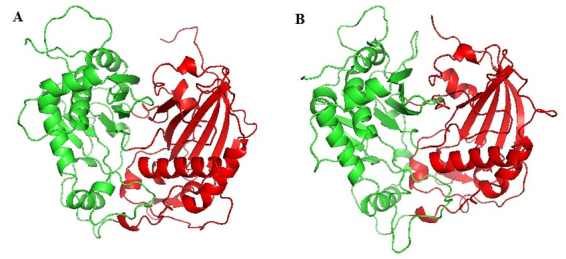
Cartoon representation for the 3-D structure of modeled proteins of *Petunia hybrida* colored per domain (Red = Domain I; Green = Domain II): (A) PhBPBT protein sequence; (B) PhCFAT protein sequence.

We inspected the final models 8 and 3 to identify the protein domains and the amino acid residues that are functionally important by aligning the models and template. The active site and the substrate-binding regions of the template were identified on the models (Table 2). We identified two domains in the template: the first domain contained amino acid residues 1-199 and 387-409, whereas the second comprised the regions between 200-386 and 410-446. Two distinct domains were also identified in the modeled PhBPBT structure (Figure 3A); domain I comprised the residues 1-204 and 376-401, whereas domain II encompassed the residues 205-375 and 402-460. Similarly, the modeled PhCFAT (Figure 3B) also showed two different domains, the first was equivalent to the residues 1-193 and 378-402, and the second domain was formed by amino acid residues 194-377 and 403-454. 

**Table 2:** Amino acid residues described as important for the protein function in template (PDB ID: 5FAL A) and their correspondent positions in the modelled PhBPBT and PhCFAT proteins.

Template (5FAL A)			BPBT (Model 8)			CFAT (Model 3)		
AA^1^	P^2^	Classification	AA^1^	P^2^	Classification	AA^1^	P^2^	Classification
Arg	369	Polar + Charged	Lys	357	Polar + Charged	Lys	357	Polar + Charged
Thr	382	Polar Uncharged	Val	370	Non-polar	Ser	373	Polar Uncharged
His	163	Polar + Charged	His	167	Polar + Charged	His	155	Polar + Charged
Trp	384	Non-polar	Asp	372	Polar - Charged	Gly	375	Non-polar
Ser	38	Polar Uncharged	Val	40	Non-polar	Gly	43	Non-polar
Tyr	40	Polar Uncharged	Asn	51	Polar Uncharged	Phe	45	Non-polar

^1^Three-letters amino acid abbreviation; ^2^Amino acid position at the protein sequence; Arg -Arginine; Thr - Threonine; His - Histidine; Trp - Tryptophan; Ser - Serine; Tyr - Tyrosine; Lys - Lysine; Val - Valine; Asp - Aspartic acid; Asn - Asparagine; Gly - Glycine; Phe - Phenylalanine. The 3D structures of PhBPBT and PhCFAT were aligned with the template structure using the PyMol software.

**Figure 4 -:**
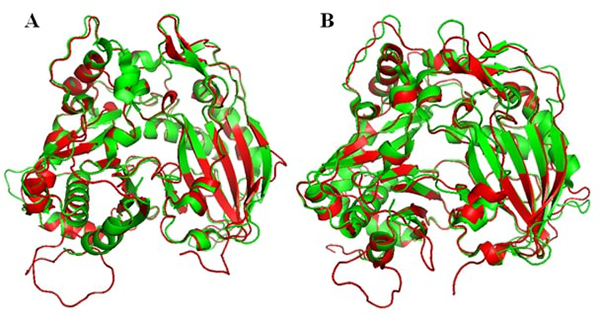
Cartoon representation for the superimposition between the 3-D *Petunia hybrida* structures and *Panicum virgatum* template: (A) PhBPBT sequence; (B) PhCFAT sequence. The template is represented in green, BPBT and CFAT in red.

**Figure 5 -:**
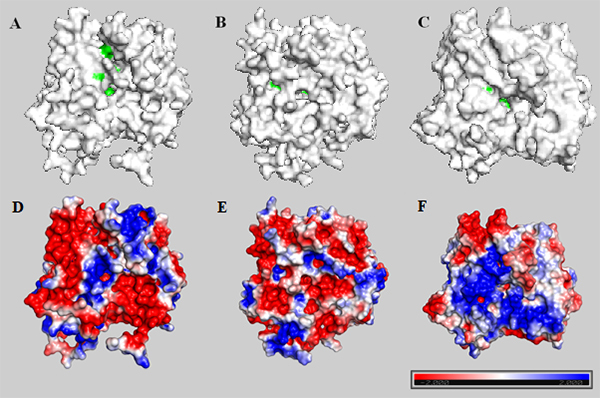
Molecular surface of PhBPBT (A, D), PhCFAT (B, E), and PvHCT2a (C, F) indicating the active sites (green) and their electrostatic potential, respectively. The most negative residues were colored in red, the neutral in white, and the most positive in blue.

The two domains had a core β-sheet surrounded by α-helices. In the PhBPBT 3D model, we found seven α-helices and nine β-sheets in domain I and nine α-helices and seven β-sheets in the second domain (Figure 3A). The modeled PhCFAT showed seven α-helices and a core containing seven β-sheets in domain I, while the domain II held ten α-helices and six β-sheets (Figure 3B). The structural alignment between the template and modeled PhBPBT (Figure 4A) and PhCFAT (Figure 4B) showed differences in their electrostatic potentials, with the most significant differences in the domain II and active site regions (Figure 5). PhBPBT and PhCFAT had pI (isoelectric point) = 3.79 and pI = 5.57, respectively. In contrast, the template PvHCT2a showed a pI = 5.52. 

In the template, the two domains had substrate-binding sites responsible for interacting with molecules that participate in the reaction catalyzed by PvHCT2a from *Panicum virgatum*, suggesting that each domain of PhCFAT and PhBPBT may have similar interactions with their respective substrates. Based on the structural alignment among models and template, we could identify the amino acid residues potentially involved in the enzyme-substrate interactions (Table 2).

We also identified some amino acid substitutions in each protein that could change the physicochemical properties of some amino acids (Table 2). In PhBPBT, the main changes were found in residues 370, 372, and 40, whereas in PhCFAT were in residues 45 and 43.

## Discussion

We described the evolutionary relationships between homologous sequences of two Solanaceae transferases involved in volatile organic compounds’ synthesis. We also modeled the tridimensional structure of these two proteins for *Petunia hybrida.*

Our results indicated that BPBT and CFAT proteins are proximately related, and both sequences of *P. hybrida*, when used as queries, recovered the same homologous protein sets. Most BPBT sequences integrated the same clade, whereas CFAT sequences grouped with other transferases from different nightshade genera. Our molecular genealogy showed proteins preferentially grouped due to their activities. In addition, several proteins seem to result from gene duplications, as many diploid species displayed multiple copies of these proteins (Figure 1; e.g., shikimate O-hydroxynamoyltransferase of *Capsicum* and *Nicotiana* that appeared in different branches in the tree). The 3D modeling reflected the structural similarity between PhBPBT and PhCFAT and indicated their functional differences.

PhBPBT and PhCFAT proteins are involved in isoeugenol biosynthesis, a phenylpropanoid volatile and prominent component of floral scent in *Petunia* (Koeduka et al., 2006). Among wild petunias, only *P. axillaris* has scented petals (Stehmann et al., 2009) and attracts hawkmoths (Venail et al., 2010). Some *P. hybrida* lineages and cultivars also produce aroma (Fenske et al., 2015), with isoeugenol as the final product in a pathway that starts with phenylalanine (Phe). In this way, CFAT acetylates coniferyl alcohol to produce coniferyl acetate (Dexter et al., 2007), and BPBT catalyzes a reaction that combines benzyl alcohol and benzoyl-CoA (Boatright et al., 2004) internally (Clark et al., 2009). The shikimic acid pathway precedes the phenylpropanoid biosynthesis (Orlova et al., 2006), and the shikimate O-hydroxycinnamoyl transferase is a crucial enzyme in this process in tobacco (Hoffmann et al., 2004). In Figure 1, we reported that PhCFAT and *Nicotiana* and *Solanum* shikimate O-hydroxycinnamoyl transferase are sister proteins, which could be explained, at least in part, by the relationships of these proteins in the same pathway that could have derived from the same ancestor by duplication and neofunctionalization, similar to other proteins that integrate a same metabolic route (e.g., Maere et al., 2005; Yockteng et al., 2013). However, our search did not recover a sequence for *P. hybrida* shikimate O-hydroxycinnamoyl transferase that was recently identified (Kim et al., 2019). However, until the PhHCT discovery, BPBT and CFAT were the only known BAHD family members in *Petunia* (Dexter *et al.,* 2007). 

We also verified some gene duplication in these proteins, even among diploid species. Many of these duplicated proteins play different functions, which suggests duplication followed by neofunctionalization in the BAHD family. Neofunctionalization due to punctual duplications in Solanaceae has been observed among other proteins (e.g., Segatto et al., 2016a,b). 

Analyzing the *Prunus mume* BAHD family (Zhang et al., 2019), the authors included PhBPBT and PhCFAT sequences in the comparisons and identified 20 conserved motifs. PhBPBT and PhCFAT showed 13 and 12 motifs, respectively, with shared order but different positions and lengths. Motif 2 contained the HxxxD, and motif 3 had the DFHWG domain, both present in all groups of sequences. In that analysis, PhBPBT and PhCFAT integrated different clades with a similar composition to that we obtained here. Other phylogenetic analyses based on BADH family sequences have also had similar results (Yu et al., 2009; Tuominen et al., 2011).

The phylogenetic position of PhBPBT and PhCFAT and their sister sequences in respective clades agree with the evolutionary relationships between genera in Solanaceae (Särkinen et al., 2013) and the time of genome origins. The *Petunia* genome was the first to diverge, followed by *Nicotiana*, *Capsicum,* and *Solanum* (Bombarely et al., 2016). This concordance suggests that multiple copies observed in other genera and not in *P. hybrida* for BPBT and CFAT were acquired posteriorly to the genera divergence and, due to *Petunia* species, in general, are not scented (Amrad et al., 2016), the copy number of each gene in the BAHD family was limited in *P. hybrida*.

Regarding the 3D models, our results showed that the modeled PhBPBT and PhCFAT proteins have a high identity (29% and 30%, respectively) and the same overall 3D fold (Figure 3) and are very similar to the *Panicum virgatum* shikimate hydroxycinnamoyl transferase. Despite the structural similarities, these three proteins differ in surface properties (Figure 4; Table 2). The three modeled proteins show two domains and some mutations in the active site that indicate differences in their interaction with the substrate and activities. 

The Arg369, Thr382, His163, Trp384, Ser38, and Tyr40 residues are implicated in the interactions with substrates in the active site of PvHCT2a (Eudes et al. 2016a). These residues corresponded to the amino acid residues Lys357, Val370, His167, Asp372, Val40, and Asn51 in the PhBPBT, whereas for PhCFAT, these PvHCT2a residues were structurally aligned to Lys357, Ser373, His155, Gly375, Gly43, and Phe45 sites. Comparing the physicochemical properties of these residues among template and models, we could evaluate the impact of the amino acid substitutions in each protein. The results indicated that some mutations in the active site region changed the physicochemical properties of some amino acids (Table 2). These structural changes observed in PhBPBT and PhCFAT proteins suggested that functional changes, such as different polarity and charges of specific amino acids, may alter functional aspects of these proteins, mainly regarding the substrate specificity.

Our results support a shared mechanism of pollinator attraction in different steps of the volatile pathway of flowers of nightshades. Identifying and characterizing these key components would provide valuable tools for future discoveries in wild species and understanding their plant-pollinator interactions.

## Supplementary material

The following online material is available for this article:

Table S1 - Protein sequences included in evolutionary analysis of volatile organic compounds in Solanaceae.
