# Supplementary material for: Molecular evolution and structural analyses of proteins involved in metabolic pathways of volatile organic compounds in Petunia hybrida (Solanaceae)
Source: Genet Mol Biol. 2022 Dec 16;46(1 Suppl 1):e20220114. doi: 10.1590/1678-4685-GMB-2022-0114 (PMC9762610; doi:10.1590/1678-4685-GMB-2022-0114)
Supplement: Table S1 - [file 1415-4757-GMB-46-1-s1-e20220114-s1.pdf]

Genetics and Molecular Biology
https://doi.org/10.1590/1678-4685-GMB-2022-0114

Supplementary Material to "Molecular evolution and structural analyses of proteins involved in metabolic pathways of volatile organic compounds in *Petunia hybrida* (Solanaceae)"

Table S1 - Protein sequences included in evolutionary analysis of volatile organic compounds in Solanaceae.

UniProt ID	Protein	Gene	Taxon	Taxon ID
Q6E593	Benzoyl coenzyme A: benzyl alcohol benzoyl transferase	PhBEBT1	*Petunia hybrida*	4102
Q8GT20	Benzyl alcohol O-benzoyltransferase	HSR201	*Nicotiana tabacum*	4097
A0A1S4C4D3	benzyl alcohol O-benzoyltransferase	LOC107814928	*Nicotiana tabacum*	4097
A0A1S3YHI1	benzyl alcohol O-benzoyltransferase-like	LOC107776214	*Nicotiana tabacum*	4097
Q43583	Hsr201 protein	hsr201	*Nicotiana tabacum*	4097
A0A1U7Z0B0	benzyl alcohol O-benzoyltransferase	LOC104249987	*Nicotiana sylvestris*	4096
A0A1J6KHR5	Benzyl alcohol o-benzoyltransferase	HSR201	*Nicotiana attenuata*	49451
A0A1U7YN93	benzyl alcohol O-benzoyltransferase-like isoform X1	LOC104248830	*Nicotiana sylvestris*	4096
A0A1S4CZW2	benzyl alcohol O-benzoyltransferase-like isoform X1	LOC107824435	*Nicotiana tabacum*	4097
A0A1U8HHZ9	benzyl alcohol O-benzoyltransferase	LOC107877868	*Capsicum annuum*	4072
A0A0V0I5E7	Putative benzyl alcohol O-benzoyltransferase-like		*Solanum chacoense*	4108
M0ZKM2	Uncharacterized protein		*Solanum tuberosum*	4113
A0A1U7YK12	benzyl alcohol O-benzoyltransferase-like	LOC104248831	*Nicotiana sylvestris*	4096
M0ZKM3	Uncharacterized protein		*Solanum tuberosum*	4113
K4CF67	Uncharacterized protein		*Solanum lycopersicum*	4081
K4CF68	Uncharacterized protein		*Solanum lycopersicum*	4081
A0A1U7YH70	benzyl alcohol O-benzoyltransferase-like isoform X2	LOC104248830	*Nicotiana sylvestris*	4096
A0A1S4CZU5	benzyl alcohol O-benzoyltransferase-like isoform X2	LOC107824435	*Nicotiana tabacum*	4097
A0A1S4CZX7	benzyl alcohol O-benzoyltransferase-like	LOC107824433	*Nicotiana tabacum*	4097
K4BYU3	Uncharacterized protein		*Solanum lycopersicum*	4081
M1B0D0	Uncharacterized protein		*Solanum tuberosum*	4113
A0A1U7UZU7	methanol O-anthraniloyltransferase-like	LOC104212170	*Nicotiana sylvestris*	4096
A0A1S3ZGZ3	methanol O-anthraniloyltransferase-like	LOC107786697	*Nicotiana tabacum*	4097
K4D716	Uncharacterized protein		*Solanum lycopersicum*	4081
A0A1U8FN24	methanol O-anthraniloyltransferase-like	LOC107857371	*Capsicum annuum*	4072
A0A1J6JV71	Methanol o-anthraniloyltransferase	AMAT_0	*Nicotiana attenuata*	49451
A0A1U8G7E5	methanol O-anthraniloyltransferase-like	LOC107862793	*Capsicum annuum*	4072
A0A1S4CTJ4	methanol O-anthraniloyltransferase-like	LOC107822411	*Nicotiana tabacum*	4097
A0A1U7WDG5	methanol O-anthraniloyltransferase-like	LOC104227468	*Nicotiana sylvestris*	4096
A0A1U8GPD3	benzyl alcohol O-benzoyltransferase-like	LOC107870838	*Capsicum annuum*	4072
A0A1U8F1B3	methanol O-anthraniloyltransferase-like	LOC107849946	*Capsicum annuum*	4072
M1CD50	Uncharacterized protein		*Solanum tuberosum*	4113
A0A1S4AB38	methanol O-anthraniloyltransferase-like	LOC107795614	*Nicotiana tabacum*	4097
A0A1U8F1A6	methanol O-anthraniloyltransferase-like	LOC107849935	*Capsicum annuum*	4072
A0A1U8FPR7	methanol O-anthraniloyltransferase-like	LOC107857372	*Capsicum annuum*	4072

UniProt ID	Protein	Gene	Taxon	Taxon ID
M1AJ88	Uncharacterized protein		*Solanum tuberosum*	4113
M1C8D7	Uncharacterized protein		*Solanum tuberosum*	4113
M1C8D6	Uncharacterized protein		*Solanum tuberosum*	4113
K4BYU6	Uncharacterized protein		*Solanum lycopersicum*	4081
K4CI65	Alcohol acyltransferase 2		*Solanum lycopersicum*	4081
A0A1U8FEN6	methanol O-anthraniloyltransferase-like	LOC107857370	*Capsicum annuum*	4072
A0A0A0RSG8	Alcohol acyltransferase 1	AAT1	*Solanum pennellii*	28526
R9R6J1	Alcohol acyltransferase 2		*Solanum pimpinellifolium*	4084
Q6QLX4	Alcohol acyl transferase	AAT1	*Solanum lycopersicum*	4081
A0A1U8FGB8	methanol O-anthraniloyltransferase-like	LOC107857374	*Capsicum annuum*	4072
M1B0D1	Uncharacterized protein		*Solanum tuberosum*	4113
K4BYU7	Uncharacterized protein		*Solanum lycopersicum*	4081
A0A0V0I5Z6	Putative omega-hydroxypalmitate O-feruloyl transferase-like		*Solanum chacoense*	4108
M1A2Q0	Uncharacterized protein		*Solanum tuberosum*	4113
K4BJG7	Uncharacterized protein		*Solanum lycopersicum*	4081
A0A1S4BKP5	omega-hydroxypalmitate O-feruloyl transferase-like	LOC107809372	*Nicotiana tabacum*	4097
A0A1U7VQR6	omega-hydroxypalmitate O-feruloyl transferase-like	LOC104220971	*Nicotiana sylvestris*	4096
A0A1S3ZLG8	omega-hydroxypalmitate O-feruloyl transferase-like	LOC107787984	*Nicotiana tabacum*	4097
A0A1U8FY47	omega-hydroxypalmitate O-feruloyl transferase-like	LOC107862886	*Capsicum annuum*	4072
M1D4C2	Uncharacterized protein		*Solanum tuberosum*	4113
D5FPG8	Feruloyl transferase		*Solanum tuberosum*	4113
A0A1S4B2P0	omega-hydroxypalmitate O-feruloyl transferase-like	LOC107803922	*Nicotiana tabacum*	4097
A0A1U7X413	omega-hydroxypalmitate O-feruloyl transferase	LOC104230174	*Nicotiana sylvestris*	4096
A0A1J6KXT0	Omega-hydroxypalmitate o-feruloyl transferase	HHT1_6	*Nicotiana attenuata*	49451
A0A1S4BLY4	omega-hydroxypalmitate O-feruloyl transferase-like	LOC107809740	*Nicotiana tabacum*	4097
K4CJ18	Uncharacterized protein		*Solanum lycopersicum*	4081
A0A1U8GB23	omega-hydroxypalmitate O-feruloyl transferase	LOC107866964	*Capsicum annuum*	4072
M1DGY6	Uncharacterized protein		*Solanum tuberosum*	4113
M0ZWL4	Uncharacterized protein		*Solanum tuberosum*	4113
A0A1U8FAS7	omega-hydroxypalmitate O-feruloyl transferase-like	LOC107852253	*Capsicum annuum*	4072
A0A1U8FEH9	omega-hydroxypalmitate O-feruloyl transferase-like	LOC107857273	*Capsicum annuum*	4072
A0A1J6IGZ3	Omega-hydroxypalmitate o-feruloyl transferase	HHT1_4	*Nicotiana attenuata*	49451
K4BV51	Uncharacterized protein		*Solanum lycopersicum*	4081
K4AX62	Uncharacterized protein		*Solanum lycopersicum*	4081
M0ZYT4	Uncharacterized protein		*Solanum tuberosum*	4113
K4BVF1	Uncharacterized protein		*Solanum lycopersicum*	4081
M1D4C3	Uncharacterized protein		*Solanum tuberosum*	4113
A0A1U7YQE0	omega-hydroxypalmitate O-feruloyl transferase	LOC104249454	*Nicotiana sylvestris*	4096
A0A1S3ZHM4	omega-hydroxypalmitate O-feruloyl transferase-like	LOC107786832	*Nicotiana tabacum*	4097
A0A1S3X8H4	3'-N-debenzoyl-2'-deoxytaxol N-benzoyltransferase-like	LOC107762258	*Nicotiana tabacum*	4097
A0A0V0I5I4	Putative 3'-N-debenzoyl-2'-deoxytaxol N-benzoyltransferase-like		*Solanum chacoense*	4108
A0A1U8ERB0	uncharacterized acetyltransferase At3g50280-like	LOC107849650	*Capsicum annuum*	4072

UniProt ID	Protein	Gene	Taxon	Taxon ID
A0A1S3ZQQ1	omega-hydroxypalmitate O-feruloyl transferase-like	LOC107789351	*Nicotiana tabacum*	4097
M1A2Q1	Uncharacterized protein		*Solanum tuberosum*	4113
A0A1S3ZP51	uncharacterized acetyltransferase At3g50280-like	LOC107788915	*Nicotiana tabacum*	4097
K4ASF6	Uncharacterized protein		*Solanum lycopersicum*	4081
Q9FXS7	EIG-I24 protein	EIG-I24	*Nicotiana tabacum*	4097
A0A1S4AUA4	omega-hydroxypalmitate O-feruloyl transferase-like	LOC107801351	*Nicotiana tabacum*	4097
E1CFE2	Acyltransferase-like		*Solanum melongena*	4111
A0A1U7WN85	omega-hydroxypalmitate O-feruloyl transferase	LOC104225952	*Nicotiana sylvestris*	4096
Q70G32	Hydroxycinnamoyl CoA quinate transferase	hqt	*Solanum lycopersicum*	4081
A0A1S4D1T0	omega-hydroxypalmitate O-feruloyl transferase-like	LOC107825023	*Nicotiana tabacum*	4097
Q9SST8	Hydroxycinnamoyl-CoA quinate hydroxycinnamoyltransferase	hcbt	*Ipomoea batatas*	4120
A0A1U7XP11	3'-N-debenzoyl-2'-deoxytaxol N-benzoyltransferase-like	LOC104238638	*Nicotiana sylvestris*	4096
A0A1U8DYB8	3'-N-debenzoyl-2'-deoxytaxol N-benzoyltransferase	LOC107840612	*Capsicum annuum*	4072
A0A1J6JT37	Omega-hydroxypalmitate o-feruloyl transferase	HHT1_0	*Nicotiana attenuata*	49451
A0A1S3ZR33	omega-hydroxypalmitate O-feruloyl transferase-like	LOC107789641	*Nicotiana tabacum*	4097
A0A1U7XYC0	omega-hydroxypalmitate O-feruloyl transferase	LOC104241380	*Nicotiana sylvestris*	4096
A0A1S4B536	omega-hydroxypalmitate O-feruloyl transferase-like	LOC107804640	*Nicotiana tabacum*	4097
M1B9W0	Uncharacterized protein		*Solanum tuberosum*	4113
A0A126Q9A5	Hydroxycinnamoyl coenzyme A-quinate transferase		*Solanum melongena*	4111
A0A1U8F1N6	spermidine coumaroyl-CoA acyltransferase	LOC107850168	*Capsicum annuum*	4072
A0A1U7WZE1	uncharacterized acetyltransferase At3g50280-like	LOC104231474	*Nicotiana sylvestris*	4096
A0A1S4AB93	shikimate O-hydroxycinnamoyltransferase-like	LOC107795706	*Nicotiana tabacum*	4097
K4CB47	Uncharacterized protein		*Solanum lycopersicum*	4081
A0A1U8H8C1	shikimate O-hydroxycinnamoyltransferase-like	LOC107877588	*Capsicum annuum*	4072
A0A1U7WTI3	3'-N-debenzoyl-2'-deoxytaxol N-benzoyltransferase	LOC104227099	*Nicotiana sylvestris*	4096
A0A1S4C523	3'-N-debenzoyl-2'-deoxytaxol N-benzoyltransferase-like	LOC107815242	*Nicotiana tabacum*	4097
K4C5Q6	Uncharacterized protein		*Solanum lycopersicum*	4081
A0A1J6IDV6	Spermidine sinapoyl-coa acyltransferase	SDT	*Nicotiana attenuata*	49451
D5M8Q1	HQT		*Solanum tuberosum*	4113
Q3HRZ5	Hydroxycinnamoyl-CoA quinate-like protein		*Solanum tuberosum*	4113
A0A1J6J702	Omega-hydroxypalmitate o-feruloyl transferase	HHT1_2	*Nicotiana attenuata*	49451
A0A1S4AUD1	agmatine coumaroyltransferase-2-like	LOC107801372	*Nicotiana tabacum*	4097
E0D875	Hydroxycinnamoyl-CoA shikimate/quinate hydroxycinnamoyltransferase	IbHCT	*Ipomoea batatas*	4120
A0A1U7WQF3	agmatine coumaroyltransferase-2-like	LOC104229805	*Nicotiana sylvestris*	4096
A0A1S3XA36	agmatine coumaroyltransferase-2-like	LOC107762886	*Nicotiana tabacum*	4097
A0A0V0I4C3	Putative shikimate O-hydroxycinnamoyltransferase-like		*Solanum chacoense*	4108
M1AS77	Uncharacterized protein		*Solanum tuberosum*	4113
A0A1U8EJS2	agmatine coumaroyltransferase-2-like	LOC107846882	*Capsicum annuum*	4072
A0A1U8H341	agmatine coumaroyltransferase-2-like isoform X1	LOC107875625	*Capsicum annuum*	4072
Q70G33	Hydroxycinnamoyl CoA quinate transferase	hqt	*Nicotiana tabacum*	4097
A0A1U7VWB9	agmatine coumaroyltransferase-2-like	LOC104220132	*Nicotiana sylvestris*	4096
M1CKC3	Uncharacterized protein		*Solanum tuberosum*	4113
A0A1S4A8Q0	agmatine coumaroyltransferase-2-like	LOC107794911	*Nicotiana tabacum*	4097
A0A1S3ZVY4	agmatine coumaroyltransferase-2-like	LOC107791075	*Nicotiana tabacum*	4097

UniProt ID	Protein	Gene	Taxon	Taxon ID
A0A1U7WKS9	agmatine coumaroyltransferase-2-like	LOC104228407	*Nicotiana sylvestris*	4096
A0A1S4D7Q6	shikimate O-hydroxycinnamoyltransferase-like isoform X2	LOC107826939	*Nicotiana tabacum*	4097
M1CY19	Uncharacterized protein		*Solanum tuberosum*	4113
A0A1U7VE63	shikimate O-hydroxycinnamoyltransferase-like	LOC104212932	*Nicotiana sylvestris*	4096
A0A1S4BFV7	shikimate O-hydroxycinnamoyltransferase-like	LOC107807845	*Nicotiana tabacum*	4097
A0A182C5N5	Hydroxycinnamoyl-CoA quinate transferase	HQT	*Nicotiana attenuata*	49451
A0A1S3Y756	shikimate O-hydroxycinnamoyltransferase-like	LOC107772942	*Nicotiana tabacum*	4097
A0A1U8FEQ1	uncharacterized acetyltransferase At3g50280-like	LOC107857394	*Capsicum annuum*	4072
K4DAK5	Uncharacterized protein		*Solanum lycopersicum*	4081
K4BLR2	Uncharacterized protein		*Solanum lycopersicum*	4081
A0A1J6IFV1	3'-n-debenzoyl-2'-deoxytaxol n-benzoyltransferase	TAX10	*Nicotiana attenuata*	49451
M1BM01	Uncharacterized protein		*Solanum tuberosum*	4113
A0A1J6I6X8	Shikimate o-hydroxycinnamoyltransferase	HST_1	*Nicotiana attenuata*	49451
A0A1S4AQ71	vinorine synthase-like	LOC107800192	*Nicotiana tabacum*	4097
A0A1U8FN03	uncharacterized acetyltransferase At3g50280-like	LOC107857340	*Capsicum annuum*	4072
A0A1S4D839	shikimate O-hydroxycinnamoyltransferase-like isoform X1	LOC107826939	*Nicotiana tabacum*	4097
B5LAV0	Putative hydroxycinnamoyl transferase	LOC107862050	*Capsicum annuum*	4072
K4CI78	Uncharacterized protein		*Solanum lycopersicum*	4081
A0A1U8FEL3	uncharacterized acetyltransferase At3g50280-like	LOC107857344	*Capsicum annuum*	4072
M1BC65	Uncharacterized protein		*Solanum tuberosum*	4113
G9HTF7	Putrescine hydroxycinnamoyl transferase	AT1	*Nicotiana attenuata*	49451
A0A1S3XSW3	shikimate O-hydroxycinnamoyltransferase-like	LOC107768362	*Nicotiana tabacum*	4097
A0A1U7Y8Z3	shikimate O-hydroxycinnamoyltransferase-like	LOC104246474	*Nicotiana sylvestris*	4096
A0A1U7Y9C4	shikimate O-hydroxycinnamoyltransferase-like	LOC104244757	*Nicotiana sylvestris*	4096
A0A1S3YVT3	pelargonidin 3-O-(6-caffeoylglucoside) 5-O-(6-O-malonylglucoside) 4'''-malonyltransferase-like	LOC107780054	*Nicotiana tabacum*	4097
A0A1U7XGZ8	vinorine synthase-like	LOC104237663	*Nicotiana sylvestris*	4096
M1B425	Uncharacterized protein		*Solanum tuberosum*	4113
A0A1S3ZTF2	salutaridinol 7-O-acetyltransferase-like	LOC107790256	*Nicotiana tabacum*	4097
A0A1U7WJQ9	salutaridinol 7-O-acetyltransferase-like	LOC104229271	*Nicotiana sylvestris*	4096
A0A1U7Y1H9	agmatine coumaroyltransferase-2-like	LOC104242452	*Nicotiana sylvestris*	4096
Q8GSM7	Shikimate O-hydroxycinnamoyltransferase	HST	*Nicotiana tabacum*	4097
A0A1S3ZBX3	vinorine synthase-like	LOC107785028	*Nicotiana tabacum*	4097
K4C9I9	Uncharacterized protein		*Solanum lycopersicum*	4081
A0A1S3Y635	BAHD acyltransferase DCR-like	LOC107772760	*Nicotiana tabacum*	4097
K4DAK4	Uncharacterized protein		*Solanum lycopersicum*	4081
M1AS76	Uncharacterized protein		*Solanum tuberosum*	4113
K4D5C3	Uncharacterized protein		*Solanum lycopersicum*	4081
M1BM02	Uncharacterized protein		*Solanum tuberosum*	4113
A0A1S3X5R7	agmatine coumaroyltransferase-2-like	LOC107761507	*Nicotiana tabacum*	4097
M1AAN1	Uncharacterized protein		*Solanum tuberosum*	4113
A0A1U8FE50	shikimate O-hydroxycinnamoyltransferase-like	LOC107854097	*Capsicum annuum*	4072
A0A1S3X5F3	agmatine coumaroyltransferase-2-like	LOC107761506	*Nicotiana tabacum*	4097
K4D9S1	Uncharacterized protein		*Solanum lycopersicum*	4081

UniProt ID	Protein	Gene	Taxon	Taxon ID
A0A1S4B614	shikimate O-hydroxycinnamoyltransferase-like	LOC107804864	*Nicotiana tabacum*	4097
A0A1J6IRT7	Shikimate o-hydroxycinnamoyltransferase	HST_6	*Nicotiana attenuata*	49451
A0A1U7V7T3	uncharacterized acetyltransferase At3g50280-like	LOC104215955	*Nicotiana sylvestris*	4096
A0A1S3ZQY7	shikimate O-hydroxycinnamoyltransferase-like	LOC107789426	*Nicotiana tabacum*	4097
A0A1J6J1I9	Shikimate o-hydroxycinnamoyltransferase	HST_5	*Nicotiana attenuata*	49451
A0A1U8FPN8	uncharacterized acetyltransferase At3g50280-like	LOC107857341	*Capsicum annuum*	4072
A0A1U7XBN2	shikimate O-hydroxycinnamoyltransferase	LOC104232368	*Nicotiana sylvestris*	4096
M1A247	Uncharacterized protein		*Solanum tuberosum*	4113
M1BM00	Uncharacterized protein		*Solanum tuberosum*	4113
A0A1U8EY07	vinorine synthase-like	LOC107851467	*Capsicum annuum*	4072
A0A1U8F4I6	omega-hydroxypalmitate O-feruloyl transferase	LOC107850816	*Capsicum annuum*	4072
A0A1S4AR82	uncharacterized acetyltransferase At3g50280-like	LOC107800579	*Nicotiana tabacum*	4097
K4C5S5	Uncharacterized protein		*Solanum lycopersicum*	4081
Q8GV04	Acyltransferase 1		*Capsicum chinense*	80379
A0A1U8DZ36	shikimate O-hydroxycinnamoyltransferase-like	LOC107840863	*Capsicum annuum*	4072
A0A1U7WG57	salutaridinol 7-O-acetyltransferase-like	LOC104223816	*Nicotiana sylvestris*	4096
A0A1U8EM36	agmatine coumaroyltransferase-2-like	LOC107848097	*Capsicum annuum*	4072
A0A1U8F0G0	uncharacterized acetyltransferase At3g50280-like	LOC107849592	*Capsicum annuum*	4072
A0A1U8GFJ4	vinorine synthase-like	LOC107865823	*Capsicum annuum*	4072
A0A1S3XAE8	shikimate O-hydroxycinnamoyltransferase-like	LOC107762853	*Nicotiana tabacum*	4097
A0A1J6IA01	Shikimate o-hydroxycinnamoyltransferase	HST_0	*Nicotiana attenuata*	49451
A0A1U8GSJ9	spermidine hydroxycinnamoyl transferase-like	LOC107871738	*Capsicum annuum*	4072
M1DXQ0	Uncharacterized protein		*Solanum tuberosum*	4113
A0A1U8ERB9	uncharacterized acetyltransferase At3g50280-like	LOC107849659	*Capsicum annuum*	4072
M1C8U8	Uncharacterized protein		*Solanum tuberosum*	4113
A0A1U8EN67	vinorine synthase-like	LOC107848376	*Capsicum annuum*	4072
A0A1S3X7N0	shikimate O-hydroxycinnamoyltransferase-like	LOC107762091	*Nicotiana tabacum*	4097
A0A1U7X8T2	shikimate O-hydroxycinnamoyltransferase-like	LOC104231954	*Nicotiana sylvestris*	4096
A0A1S4AKH0	acylsugar acyltransferase 3-like	LOC107798604	*Nicotiana tabacum*	4097
A0A1J6IBI0	Pelargonidin 3-o-(6-caffeoylglucoside) 5-o-(6-o-malonylglucoside) 4'''-malonyltransferase	5MAT2_2	*Nicotiana attenuata*	49451
M1BMT6	Uncharacterized protein		*Solanum tuberosum*	4113
A0A1U8FPS7	uncharacterized acetyltransferase At3g50280-like	LOC107857392	*Capsicum annuum*	4072
M1DEJ6	Uncharacterized protein		*Solanum tuberosum*	4113
A0A1U8FPQ2	uncharacterized acetyltransferase At3g50280-like	LOC107857352	*Capsicum annuum*	4072
A0A1U7XAA9	shikimate O-hydroxycinnamoyltransferase-like	LOC104234685	*Nicotiana sylvestris*	4096
A0A1S4D2P5	uncharacterized acetyltransferase At3g50280-like	LOC107825276	*Nicotiana tabacum*	4097
A0A1S3ZFY1	shikimate O-hydroxycinnamoyltransferase-like	LOC107786308	*Nicotiana tabacum*	4097
M1C5Q7	Uncharacterized protein		*Solanum tuberosum*	4113
**A1XWY7**	**Coniferyl alcohol acyltransferase**	**CFAT**	***Petunia hybrida***	**4102**
K4BF05	Alcohol acyl transferase	AAT2	*Solanum lycopersicum*	4081
M0ZHZ9	Uncharacterized protein		*Solanum tuberosum*	4113
A0A1U8EH79	vinorine synthase	LOC107846800	*Capsicum annuum*	4072
I6TSB7	AT1	AT1	*Solanum lycopersicum*	4081
A0A1S3XX61	vinorine synthase-like	LOC107769729	*Nicotiana tabacum*	4097
A0A1U7W175	vinorine synthase-like	LOC104223945	*Nicotiana sylvestris*	4096

UniProt ID	Protein	Gene	Taxon	Taxon ID
K4CCB3	Uncharacterized protein		*Solanum lycopersicum*	4081
A0A1S3XS09	vinorine synthase-like	LOC107768066	*Nicotiana tabacum*	4097
A0A1U7W0A2	vinorine synthase-like	LOC104223692	*Nicotiana sylvestris*	4096
A0A1U8G0C4	BAHD acyltransferase DCR	LOC107863241	*Capsicum annuum*	4072
A0A1J6IKE7	Acylsugar acyltransferase 3	ASAT3_15	*Nicotiana attenuata*	49451
A0A1S4AL88	vinorine synthase-like	LOC107798845	*Nicotiana tabacum*	4097
A0A1S3ZXD3	agmatine coumaroyltransferase-2-like	LOC107791446	*Nicotiana tabacum*	4097
Q9MBD4	Acyltransferase homolog	PAT48	*Petunia hybrida*	4102
A0A1U7V7T9	uncharacterized acetyltransferase At3g50280-like	LOC104214485	*Nicotiana sylvestris*	4096
A0A1J6JYD2	Acylsugar acyltransferase 3	ASAT3_6	*Nicotiana attenuata*	49451
A0A1U8F2L9	spermidine hydroxycinnamoyl transferase	LOC107853210	*Capsicum annuum*	4072
A0A1U7WKW9	vinorine synthase-like	LOC104229643	*Nicotiana sylvestris*	4096
M0ZXU6	Uncharacterized protein		*Solanum tuberosum*	4113
A0A1S4B5J3	vinorine synthase-like	LOC107804712	*Nicotiana tabacum*	4097
A0A1U7WF38	vinorine synthase-like	LOC104227901	*Nicotiana sylvestris*	4096
K4B6F9	Uncharacterized protein		*Solanum lycopersicum*	4081
K4DH98	Uncharacterized protein		*Solanum lycopersicum*	4081
A0A1U8G8K7	vinorine synthase-like	LOC107865992	*Capsicum annuum*	4072
M4I0T7	Alcohol acyltransferase	AAT1	*Physalis peruviana*	126903
A0A1S4C6D9	uncharacterized acetyltransferase At3g50280-like isoform X1	LOC107815684	*Nicotiana tabacum*	4097
A0A1S4BLB8	shikimate O-hydroxycinnamoyltransferase-like	LOC107809545	*Nicotiana tabacum*	4097
A0A1U7YAP7	shikimate O-hydroxycinnamoyltransferase-like	LOC104245147	*Nicotiana sylvestris*	4096
M1AAQ0	Uncharacterized protein		*Solanum tuberosum*	4113
K4BYF3	Uncharacterized protein		*Solanum lycopersicum*	4081
M1BGV8	Uncharacterized protein		*Solanum tuberosum*	4113
K4B9H2	Uncharacterized protein		*Solanum lycopersicum*	4081
M1AMV7	Uncharacterized protein		*Solanum tuberosum*	4113
K4BDB4	Uncharacterized protein		*Solanum lycopersicum*	4081
A0A1S3YB63	vinorine synthase-like	LOC107774364	*Nicotiana tabacum*	4097
A0A1S4C8P4	vinorine synthase-like	LOC107816321	*Nicotiana tabacum*	4097
M1D3L8	Uncharacterized protein		*Solanum tuberosum*	4113
A0A1J6IIH2	Acylsugar acyltransferase 3	ASAT3_13	*Nicotiana attenuata*	49451
A0A1S4CEU5	vinorine synthase-like	LOC107818220	*Nicotiana tabacum*	4097
A0A1U7XKK7	spermidine hydroxycinnamoyl transferase	LOC104235054	*Nicotiana sylvestris*	4096
A0A1S4D8N2	spermidine hydroxycinnamoyl transferase-like isoform X1	LOC107827166	*Nicotiana tabacum*	4097
A0A1S4AW47	acylsugar acyltransferase 3-like	LOC107801995	*Nicotiana tabacum*	4097
A0A1U8GT83	vinorine synthase-like	LOC107872217	*Capsicum annuum*	4072
E1CFE3	Acyltransferase-like		*Solanum melongena*	4111
A0A1S3X3U6	vinorine synthase-like	LOC107760962	*Nicotiana tabacum*	4097
A0A1U8GIA0	acetyl-CoA-benzylalcohol acetyltransferase-like	LOC107866160	*Capsicum annuum*	4072
A0A1S4DDX9	vinorine synthase-like	LOC107828764	*Nicotiana tabacum*	4097
A0A1U7XL06	vinorine synthase-like	LOC104237779	*Nicotiana sylvestris*	4096
A0A1S4BEQ4	shikimate O-hydroxycinnamoyltransferase-like	LOC107807517	*Nicotiana tabacum*	4097

UniProt ID	Protein	Gene	Taxon	Taxon ID
A0A1U8H6X5	vinorine synthase-like	LOC107876947	*Capsicum annuum*	4072
K4C1U9	Uncharacterized protein		*Solanum lycopersicum*	4081
A0A0V0I4R5	Putative shikimate O-hydroxycinnamoyltransferase-like		*Solanum chacoense*	4108
K4BA44	Uncharacterized protein		*Solanum lycopersicum*	4081
A0A1S3XKH2	vinorine synthase-like	LOC107765976	*Nicotiana tabacum*	4097
A0A1J6JQ68	Acylsugar acyltransferase 3	ASAT3_0	*Nicotiana attenuata*	49451
A0A1S4C6T9	uncharacterized acetyltransferase At3g50280-like isoform X2	LOC107815684	*Nicotiana tabacum*	4097
A0A1U8HGH4	vinorine synthase-like	LOC107877439	*Capsicum annuum*	4072
A0A1U8FPA1	vinorine synthase-like	LOC107857165	*Capsicum annuum*	4072
A0A1U8FI76	acylsugar acyltransferase 3-like	LOC107858255	*Capsicum annuum*	4072
M1BCL2	Uncharacterized protein		*Solanum tuberosum*	4113
A0A1U8EZ82	acylsugar acyltransferase 3-like	LOC107851983	*Capsicum annuum*	4072
A0A1U8FSQ1	acylsugar acyltransferase 3-like	LOC107861362	*Capsicum annuum*	4072
C1JZ72	Acyltransferase (Fragment)	AT3-1	*Solanum melongena*	4111
D7F4B0	BAHD acyltransferase (Fragment)	AT3	*Capsicum chacoense*	107817
A0A1U7VMT7	vinorine synthase-like	LOC104215130	*Nicotiana sylvestris*	4096
A0A1S4AFT2	vinorine synthase-like	LOC107797201	*Nicotiana tabacum*	4097
A0A1S3XJY8	vinorine synthase-like	LOC107766049	*Nicotiana tabacum*	4097
A0A1U8GCG6	anthranilate N-benzoyltransferase protein 1	LOC107864010	*Capsicum annuum*	4072
A0A1S4CX28	vinorine synthase-like	LOC107823446	*Nicotiana tabacum*	4097
A0A1S3X3I2	anthranilate N-benzoyltransferase protein 1-like	LOC107760889	*Nicotiana tabacum*	4097
M1A248	Uncharacterized protein		*Solanum tuberosum*	4113
A0A1S3XJT4	(Z)-3-hexen-1-ol acetyltransferase-like	LOC107766042	*Nicotiana tabacum*	4097
K4CC78	Uncharacterized protein		*Solanum lycopersicum*	4081
M1A249	Uncharacterized protein		*Solanum tuberosum*	4113
M1A250	Uncharacterized protein		*Solanum tuberosum*	4113
M1A246	Uncharacterized protein		*Solanum tuberosum*	4113
K4CCE8	Uncharacterized protein		*Solanum lycopersicum*	4081
A0A1U7VW05	anthranilate N-benzoyltransferase protein 1	LOC104217551	*Nicotiana sylvestris*	4096
A0A1S4DKX5	anthranilate N-benzoyltransferase protein 1-like	LOC107830875	*Nicotiana tabacum*	4097
A0A1J6IFF4	Omega-hydroxypalmitate o-feruloyl transferase	HHT1_5	*Nicotiana attenuata*	49451
A0A1U8GTR0	acylsugar acyltransferase 3-like	LOC107872282	*Capsicum annuum*	4072
A0A1U7YDC9	vinorine synthase-like	LOC104243327	*Nicotiana sylvestris*	4096
A0A1S4DN57	vinorine synthase-like	LOC107831590	*Nicotiana tabacum*	4097
A0A1U7XYX9	agmatine coumaroyltransferase-2-like	LOC104242737	*Nicotiana sylvestris*	4096
K4BHP3	Uncharacterized protein		*Solanum lycopersicum*	4081
A0A1S4AL82	acylsugar acyltransferase 3-like	LOC107798964	*Nicotiana tabacum*	4097
K4C1A8	Uncharacterized protein		*Solanum lycopersicum*	4081
K4BPC3	Uncharacterized protein		*Solanum lycopersicum*	4081
A0A1S4DAA9	acetyl-CoA-benzylalcohol acetyltransferase-like	LOC107827656	*Nicotiana tabacum*	4097
A0A1U8FC87	acylsugar acyltransferase 3-like	LOC107855820	*Capsicum annuum*	4072
M1BAY5	Uncharacterized protein		*Solanum tuberosum*	4113
M1ADM6	Uncharacterized protein		*Solanum tuberosum*	4113

UniProt ID	Protein	Gene	Taxon	Taxon ID
K4C1U8	Uncharacterized protein		*Solanum lycopersicum*	4081
A0A1U7X3W7	uncharacterized acetyltransferase At3g50280	LOC104230129	*Nicotiana sylvestris*	4096
A0A1S3ZVB7	uncharacterized acetyltransferase At3g50280-like	LOC107790881	*Nicotiana tabacum*	4097
M1D815	Uncharacterized protein		*Solanum tuberosum*	4113
A0A1U8G2B2	acylsugar acyltransferase 3-like	LOC107861279	*Capsicum annuum*	4072
A0A1J6IJA5	Bahd acyltransferase	BAHD1_3	*Nicotiana attenuata*	49451
M1CFM8	Uncharacterized protein		*Solanum tuberosum*	4113
A0A0V0I5Q6	Putative BAHD acyltransferase DCR-like		*Solanum chacoense*	4108
A0A1U8FCQ1	uncharacterized acetyltransferase At3g50280-like	LOC107853000	*Capsicum annuum*	4072
K4AT28	Uncharacterized protein		*Solanum lycopersicum*	4081
A0A1J6IEG5	Putative acetyltransferase	A4A49_04674	*Nicotiana attenuata*	49451
A0A0U3J614	Acylsugar acyltransferase 2	ASAT2	*Solanum pennellii*	28526
K4CEM4	Uncharacterized protein		*Solanum lycopersicum*	4081
A0A1S4ARY5	uncharacterized acetyltransferase At3g50280-like	LOC107800644	*Nicotiana tabacum*	4097
K4BA47	Uncharacterized protein		*Solanum lycopersicum*	4081
A0A1U7W8X2	vinorine synthase-like	LOC104225026	*Nicotiana sylvestris*	4096
A0A1S4CVE7	acylsugar acyltransferase 3-like	LOC107823015	*Nicotiana tabacum*	4097
M1BCN4	Uncharacterized protein		*Solanum tuberosum*	4113
A0A1U8GL24	uncharacterized acetyltransferase At3g50280-like	LOC107867174	*Capsicum annuum*	4072
Q8LLM2	AER		*Nicotiana tabacum*	4097
A0A1U8H0H1	acylsugar acyltransferase 3-like	LOC107874718	*Capsicum annuum*	4072
A0A0U3J8D4	Acylsugar acyltransferase 2	ASAT2	*Solanum habrochaites*	62890
A0A1U8GU10	uncharacterized acetyltransferase At3g50280-like	LOC107872616	*Capsicum annuum*	4072
M1AXR3	Uncharacterized protein		*Solanum tuberosum*	4113
A0A1U8G9K4	acetyl-CoA-benzylalcohol acetyltransferase-like	LOC107866161	*Capsicum annuum*	4072
A0A1J6KS29	Putative acetyltransferase	A4A49_32088	*Nicotiana attenuata*	49451
A0A1S4BF37	uncharacterized acetyltransferase At3g50280-like	LOC107807544	*Nicotiana tabacum*	4097
A0A0V0I3V7	Putative deacetylvindoline O-acetyltransferase-like		*Solanum chacoense*	4108
M1DC08	Uncharacterized protein		*Solanum tuberosum*	4113
A0A1J6ING3	Bahd acyltransferase	BAHD1_9	*Nicotiana attenuata*	49451
A0A1S4C2F3	uncharacterized acetyltransferase At3g50280-like	LOC107814440	*Nicotiana tabacum*	4097
A0A1U7W573	uncharacterized acetyltransferase At3g50280	LOC104225117	*Nicotiana sylvestris*	4096
M1CLC0	Uncharacterized protein		*Solanum tuberosum*	4113
A0A1S3ZTH9	acylsugar acyltransferase 3-like	LOC107790248	*Nicotiana tabacum*	4097
A0A1U8EG32	BAHD acyltransferase DCR	LOC107842775	*Capsicum annuum*	4072
M1BTC9	Uncharacterized protein		*Solanum tuberosum*	4113
A0A1U8FM12	acylsugar acyltransferase 3-like	LOC107859692	*Capsicum annuum*	4072
A0A0U3HYV2	Acylsugar acyltransferase 2	ASAT2	*Solanum galapagense*	315350
A0A0U3JIQ9	Acylsugar acyltransferase 2	ASAT2	*Solanum pimpinellifolium*	4084
K4BPQ4	Acylsugar acyltransferase 2	ASAT2	*Solanum lycopersicum*	4081
